# Photocurable ABA triblock copolymer-based ion gels utilizing photodimerization of coumarin†

**DOI:** 10.1039/c7ra13181j

**Published:** 2018-01-17

**Authors:** Ryota Tamate, Takeshi Ueki, Aya Mizutani Akimoto, Ryo Yoshida, Toshiyuki Oyama, Hisashi Kokubo, Masayoshi Watanabe

**Affiliations:** Department of Chemistry and Biotechnology, Yokohama National University 79-5 Tokiwadai, Hodogaya-ku Yokohama 240-8501 Japan mwatanab@ynu.ac.jp; National Institute for Materials Science (NIMS) 1-1 Namiki Tsukuba Ibaraki 305-0044 Japan; Department of Materials Engineering School of Engineering, The University of Tokyo 7-3-1 Hongo, Bunkyo-ku Tokyo 113-8656 Japan

## Abstract

Herein, we develop a photocurable ABA triblock copolymer-based ion gel, which can be converted from a thermally processable, physically crosslinked ion gel to a thermally and mechanically stable, chemically crosslinked ion gel *via* photoinduced dimerization. The A block consists of a random copolymer of *N*-isopropylacrylamide and a coumarin-containing acrylate monomer, while the B block consists of an ionic liquid-philic poly(ethylene oxide). Due to the upper critical solution temperature-type phase behavior of the A block, the ABA triblock copolymer undergoes gel-to-sol transitions in a hydrophobic ionic liquid as the temperature is increased. Furthermore, under ultraviolet (UV) light irradiation, the physical crosslinks formed by association of the A blocks in the gel at low temperatures become chemically crosslinked as a result of photodimerization of the coumarin moieties in the A block; this results in conversion from a thermo-reversible, physically crosslinked ion gel to a thermo-irreversible, chemically crosslinked ion gel. The rheological changes of the ion gel upon UV irradiation have been investigated in detail. In addition, photopatterning of the ion gel has been realized by exploiting the photocurable behavior of the ABA triblock copolymer in the ionic liquid.

## Abstract 2

Photoinduced dimerization of coumarin was utilized to develop a photocurable ABA triblock copolymer-based ion gel.

## Graphical abstract

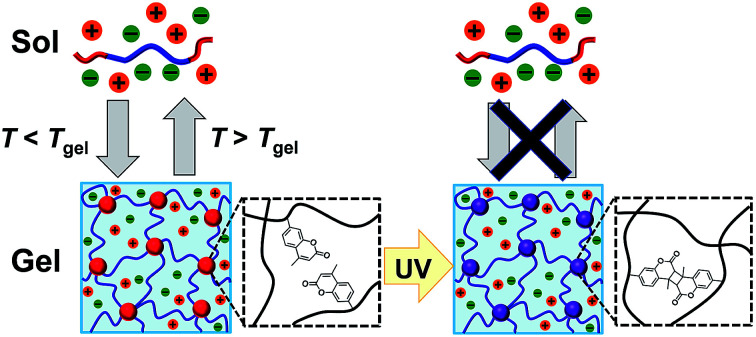

## Introduction

Ion gels are three-dimensional molecular networks swollen by room-temperature ionic liquids (ILs).^1–3^ Compared with conventional hydrogels and organogels, ion gels have unique properties that are attributable to the inherent properties of ILs; these include non-volatility, non-flammability and high ionic conductivity. These unique properties make ion gels potentially useful solid electrolytes for electrochemical devices such as electric double-layer capacitors,^4–6^ lithium-ion batteries^7,8^ and soft actuators.^9–12^ Facile processing of ion gels is required for device fabrication. Thermoresponsive ion gels that exhibit sol–gel transitions are of great interest because liquid-state processing is possible by controlling the temperature. Lodge *et al.* first demonstrated thermoresponsive ion gels based on ABA triblock copolymers.^13^ The thermoresponsive A block consisted of poly(*N*-isopropylacrylamide) (PNIPAm), which shows upper critical solution temperature (UCST) phase behaviour in ILs,^14^ whereas the B block consisted of IL-philic poly(ethylene oxide) (PEO). At low temperatures, the A block is insoluble in ILs and aggregates to form a micellar core. Above the chain overlap polymer concentration, the micellar core formed by the association of A blocks is bridged by IL-philic PEO blocks, resulting in a percolated micellar network (*i.e.*, a gel state). At high temperatures above the UCST-type phase transition temperature, the associated A blocks become soluble in ILs. Consequently, gel-to-sol transition is observed with increasing temperature. This low-temperature gel/high-temperature sol behaviour provides easy handling, enabling liquid processing at high temperatures without the use of additional solvents; furthermore, a solid form is maintained during low-temperature use.

One of the drawbacks of thermoresponsive gels based on ABA triblock copolymers is the mechanical integrity. As the association of A blocks is a transient physical crosslink, there is a finite relaxation time.^15^ Therefore, over a sufficiently long timescale, the physical crosslinks can relax and creep flow can occur.^16,17^ In addition, when the sol–gel transition temperature is near the operating temperature range, the mechanical properties of the gel are susceptible to subtle temperature fluctuations. On the other hand, a higher sol–gel transition temperature necessitates a high processing temperature that is unsafe and entails high energy consumption.

**Fig. 1:**
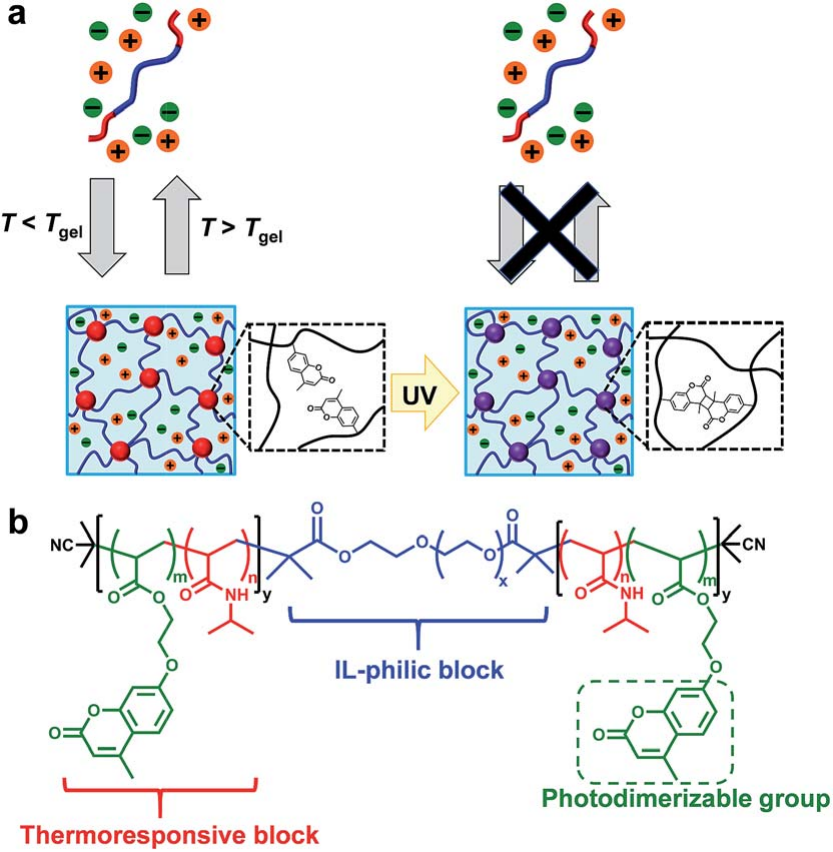
(a) Schematic illustration of an ABA triblock copolymer-based photocurable ion gel. (b) Chemical structure of the ABA triblock copolymer.

To improve the mechanical stability of physically crosslinked gels, post-chemical crosslinking of physically crosslinked gels have been developed for both hydrogel^18–21^ and ion-gel systems.^22,23^ Seminal work by Lodge *et al.* demonstrated the chemical crosslinking of ABA triblock copolymer-based ion gels by the introduction of azide-functionalized styrene units into the IL-phobic A block.^22,23^ We have also previously reported thermo/photoresponsive ABA triblock copolymer-based hydrogels whose viscoelasticity could be dynamically modulated by photodimerization of coumarin moieties introduced into the A block, resulting in a significant effect on the proliferation behaviour of encapsulated cells.^21^ Herein, our previously developed photo/thermoresponsive ABA triblock copolymer has been extended to an IL system for the first time. We demonstrate ABA triblock copolymer-based photocurable ion gels in which temperature-dependent sol–gel transitions attributed to the UCST nature of the A block in ILs were observed before UV irradiation. After UV light irradiation, we obtained photodimerization-induced chemically crosslinked ion gels that maintained their gel state irrespective of temperature (Fig. 1a).

## Results and discussion

The ABA triblock copolymer used in this study was composed of a photo/thermoresponsive A block and an IL-philic B block. The A block is a random copolymer of *N*-isopropylacrylamide (NIPAm) and 7-(2-acryloyloxyethoxy)-4-methylcoumarin (coumarin acrylate; CA), and the B block is IL-philic PEO (Fig. 1b). The coumarin units, which are known to undergo [2 + 2] photocycloaddition when exposed to UV light,^24,25^ were introduced into the A block to chemically crosslink the micellar core. The ABA triblock copolymer, P(NIPAm-*r*-CA)-*b*-PEO-*b*-P(NIPAm-*r*-CA), was successfully synthesized by reversible addition-fragmentation chain transfer (RAFT) polymerization of NIPAm and CA with PEO macro chain transfer agent (PEO-CTA) (Scheme S1, Fig. S1 and S2†). The results of polymer characterization are summarized in Table 1. The molar ratio of NIPAm and CA was set at 94.5 : 5.5. In this study, the ABA triblock copolymer was dissolved in a hydrophobic IL, 1-butyl-3-methylimidazolium hexafluorophosphate ([C_4_mim]PF_6_), in which PNIPAm is known to undergo UCST phase transition.^26^

**Table 1:** Characterization results for PEO-CTA and the ABA triblock copolymer

	*M*_n_ a (kDa)	PDI^b^	[CA]/[NIPAm]^c^ (mol%)
PEO-CTA	35.0	1.08	
ABA	17.5–35.0–17.5	1.36	5.5/94.5

a Number-averaged molecular weight calculated from ^1^H nuclear magnetic resonance (NMR).

b Polydispersity index (PDI) measured using gel permeation chromatography (GPC).

c Molar ratio of CA over NIPAm measured using ^1^H NMR.

**Fig. 2:**
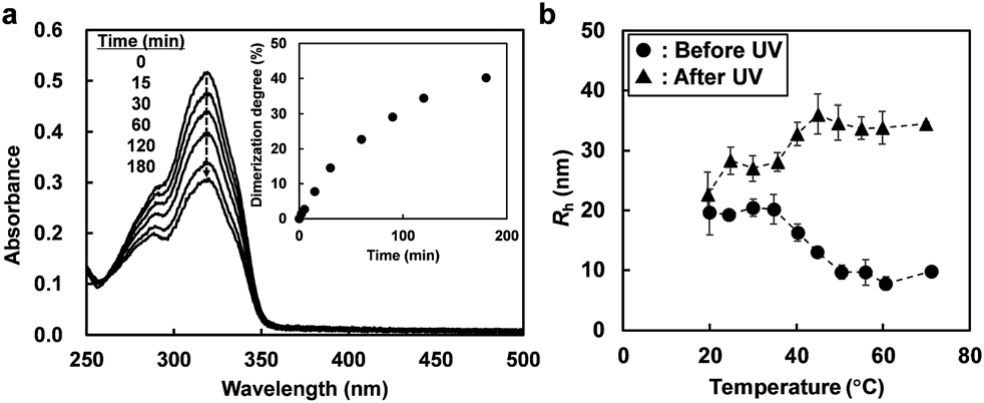
(a) Change in the UV-vis spectra of a solution of 0.1 wt% ABA triblock copolymer in [C_4_mim]PF_6_ under UV light (*λ* = 365 nm, 20 mW cm^−2^). (Inset) change in the degree of dimerization observed under UV light. (b) Temperature dependence of *R*_h_ values for the 1 wt% ABA triblock copolymer in [C_4_mim]PF_6_ before and after UV irradiation for 2 h.

The photosensitivity of a dilute solution of the ABA triblock copolymer in [C_4_mim]PF_6_ was investigated by UV-vis spectroscopy. Fig. 2a shows the time-dependent UV-vis spectra of a solution of 0.1 wt% ABA triblock copolymer in [C_4_mim]PF_6_ under UV irradiation (*λ* = 365 nm, 20 mW cm^−2^). The characteristic absorbance peak at 320 nm is ascribed to undimerized coumarin moieties introduced into the ABA triblock copolymer. The absorbance peak decreases under UV irradiation; this indicates that the photodimerization reaction of the coumarin moieties proceeds in [C_4_mim]PF_6_. The dimerization degree of the coumarin units can be calculated from the decrease in the intensity of the absorbance peak (Fig. 2a, inset). The reaction rate of photodimerization in [C_4_mim]PF_6_ is relatively slow compared to that in water.^21,27,28^ This could be explained by the high viscosity of the ILs, which retards the intermolecular collision probability. However, taking into account the fact that a number of coumarin units are incorporated into one A block (8 molecules on average, from ^1^H NMR data), the majority of A blocks are considered to be crosslinked even at a low degree of dimerization.

The temperature dependence of the hydrodynamic radius (*R*_h_) of 1 wt% ABA triblock copolymer in [C_4_mim]PF_6_ before and after UV irradiation was investigated by dynamic light scattering (DLS) measurements (Fig. 2b). Before UV light irradiation, the *R*_h_ value decreases from around 20 nm (<35 °C) to below 10 nm (>50 °C) with increasing temperature; this could be attributed to upper critical micellization temperature (UCMT) behaviour of the ABA triblock copolymer. On the other hand, after UV light irradiation at 30 °C, the *R*_h_ value increases at higher temperatures. Under UV irradiation, the coumarin moieties present in the micelle cores form [2 + 2] cyclobutane rings that act as chemical crosslinking points. Therefore, the permanent chemical crosslinks inhibit the micelle-to-unimer transition that occurs upon heating. Instead, at high temperature, the crosslinked A blocks became IL-philic and an increase in *R*_h_ is induced as the micelle core is swollen by [C_4_mim]PF_6_. It should be noted that after UV light irradiation, a slight increase in *R*_h_ values, even at low temperatures below the UCMT, and a broader distribution of *R*_h_ values (reduced second cumulant values of *μ*_2_/*Γ*^2^ = 0.32 and 1.31 at 30 °C before and after UV irradiation, respectively) were observed, suggesting that not only intramicellar but also intermicellar chemical crosslinking has occurred. Actually, a small quantity of large aggregates attributed to the intermicellar bridging in addition to small spherical micelles was confirmed by cryogenic transmission electron microscopy for ABA triblock copolymers showing UCMT behaviour in ILs.^29^

**Fig. 3:**
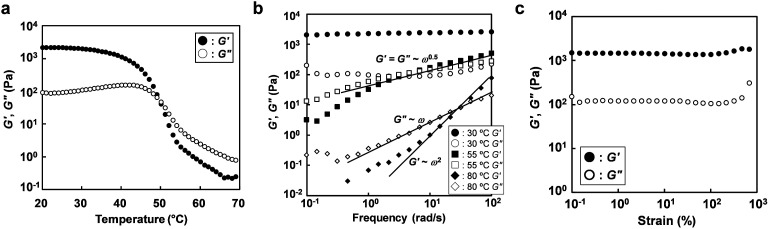
(a) Temperature dependence (1 rad s^−1^), (b) frequency dependence at different temperatures and (c) strain dependence (1 rad s^−1^) of storage (*G*′) and loss (*G*′′) moduli for a solution of 5 wt% ABA triblock copolymer in [C_4_mim]PF_6_.

With increasing polymer concentration, micelle cores formed by association of P(NIPAm-*r*-CA) blocks were bridged by IL-philic PEO blocks, leading to a percolating micellar network. Disordered micellar structures for ABA triblock copolymer-based ion gels were previously observed by atomic force microscope measurements.^10^ From qualitative inversion tests, we found that ion gels were formed at room temperature at a polymer concentration as low as 3 wt% (Fig. S3†). Fig. 3a shows the variation of the storage (*G*′) and loss (*G*′′) moduli of a 5 wt% ABA triblock copolymer dissolved in [C_4_mim]PF_6_ upon temperature changes without UV irradiation. At low temperature, *G*′ is higher than *G*′′, indicating the gel state. When the temperature is increased, both *G*′ and *G*′′ decrease; finally, *G*′′ becomes higher than *G*′ (*i.e.*, the sol state). This could be explained by the fact that percolated micellar networks are dissociated to unimers due to the UCST-type phase behaviour of the P(NIPAm-*r*-CA) block. The gel-to-sol transition temperature (*T*_gel_), defined as the crossover of *G*′ and *G*′′, was determined to be 50.1 °C. Consistent with the temperature sweep measurements, frequency sweep measurements (Fig. 3b) indicate that the polymer solution is in the gel state at 30 °C, with a *G*′ larger than *G*′′ over the entire frequency range (*ω* = 0.1–100 rad s^−1^); in contrast, terminal flow behaviour (*G*′ ∝ *ω*^2^ and *G*′′ ∝ *ω*) indicative of a liquid state was observed at 80 °C. At an intermediate temperature (55 °C) near the *T*_gel_ determined by the temperature sweep measurement, both *G*′ and *G*′′ show similar power-law behaviour (*G*′ ∼ *G*′′ ∝ *ω*^0.5^). Strain resistance of the ion gel at 30 °C was also investigated, as shown in Fig. 3c. The ion gels show linear rheological responses (*i.e.*, *G*′ and *G*′′ are not strain dependent) within the strain range of 0.1 to 200%; even at 1000% strain, breakdown of the gel network was not observed.

**Fig. 4:**
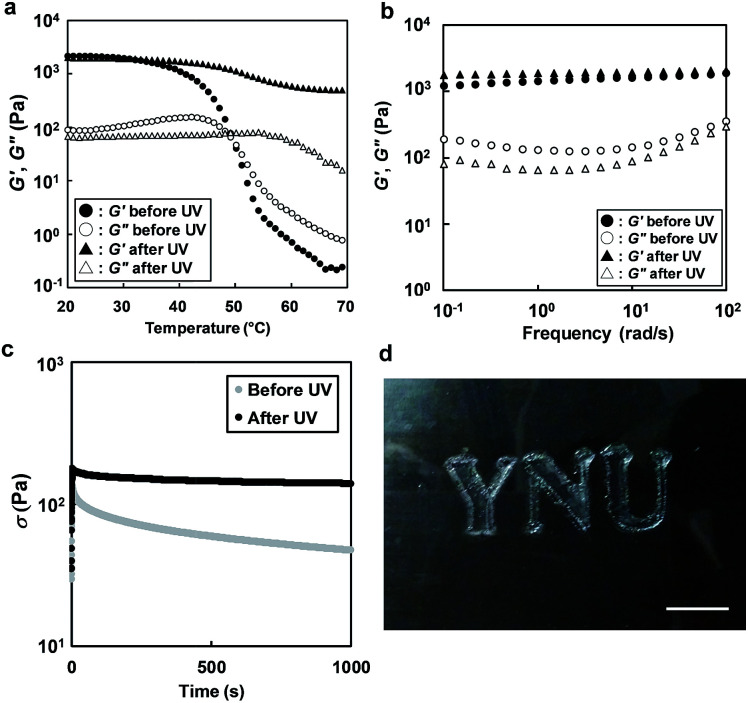
(a) Temperature dependence of *G*′ and *G*′′ for the 5 wt% ABA triblock copolymer in [C_4_mim]PF_6_ before and after UV irradiation (*λ* = 365 nm, 20 mW cm^−2^, 2 h) at a frequency of 1 rad s^−1^ and a strain of 1%. (b and c) Frequency dependence of *G*′ and *G*′′ (b) and stress relaxation measurements (c) at 30 °C for the ion gel (5 wt% ABA triblock copolymer in [C_4_mim]PF_6_) before and after UV irradiation. (d) Photograph of the patterned ion gel at room temperature. Scale bar: 1 cm.

The effect of UV light irradiation on the rheological properties of an ion gel composed of 5 wt% ABA triblock copolymer in [C_4_mim]PF_6_ was investigated. During UV light irradiation at 30 °C, a continuous increase in *G*′ and decrease in *G*′′ were observed (Fig. S4†). Incidentally, the loss tangent (tan *δ* = *G*′′/*G*′) decreased upon UV irradiation. This clearly suggests that the stiffening of the ion gel was induced by photodimerization of the coumarin moieties in the micelle cores. Time variation of UV-vis spectra for the ion gel under UV irradiation was also investigated (Fig. S5†). The dimerization degree of coumarin moieties in the ion gel reached 40% after 2 h, which was slightly higher than that in the diluted polymer solution shown in Fig. 2a. This could be explained by the increased collision probability of coumarin moieties in the concentrated system. The temperature dependence of *G*′ and *G*′′ for the ABA triblock copolymer solution in [C_4_mim]PF_6_ before and after UV irradiation is shown in Fig. 4a. After UV irradiation, the gel-to-sol transition upon heating is no longer observed, and a gel-like consistency was maintained over the temperature range of 20–70 °C. Slight decreases in the values of *G*′ and *G*′′ were observed around *T*_gel_, which implies softening of the crosslinked micelle core due to swelling with [C_4_mim]PF_6_. Frequency sweep and stress relaxation measurements were conducted at 30 °C to investigate the differences between the rheological behaviour before and after UV irradiation in detail (Fig. 4b and c). From the frequency sweep measurements (Fig. 4b), we found that the difference in *G*′ is more pronounced in the low frequency region. In addition, the stress relaxation tests (Fig. 4c) reveal that the long-term rheological behaviour has been significantly modulated by UV light irradiation. Before UV light irradiation, significant stress relaxation is observed, and half of the initial stress is relaxed after about 300 s. On the other hand, after UV light irradiation, the ion gel retains 80% of the initial stress, even after 1000 s. The marked difference in the stress relaxation behaviour could be attributable to the different types of crosslinks.^30^ Before UV irradiation, the crosslinks of the ion gel are formed by physical association of the A blocks; these crosslinks have a finite relaxation time. After UV irradiation, photodimerization of the coumarin moieties occurs within the association of the A blocks, converting the reversible physical crosslinks to permanent chemical crosslinks that have an infinite relaxation time. In contrast to the significant rheological changes, the ionic conductivity before and after UV irradiation is almost unchanged, suggesting that the photodimerization reaction in the associated A block did not affect the ionic mobility (Fig. S6†).

Finally, photopatterning of the ion gel was demonstrated by exploiting the photo-curing feature of the ion gel. The 5 wt% ABA triblock copolymer in [C_4_mim]PF_6_ was cast onto a glass slide, forming an ion-gel film at room temperature. Then, the ion gel was irradiated with UV light through a photomask. The UV-irradiated ion gel was rinsed in chloroform, and the non-irradiated area (*i.e.*, the physically crosslinked ion gel) was removed to obtain a patterned ion gel (Fig. 4d). As expected, the patterned ion gel maintained its shape integrity, even at temperatures above *T*_gel_.

## Conclusions

In conclusion, we fabricated a photocurable ion gel based on an ABA triblock copolymer. Before UV light irradiation, the ABA triblock copolymer in the hydrophobic IL showed reversible sol–gel transitions in response to temperature changes, due to the UCST-type solubility change of the A blocks. In contrast, after UV light irradiation, the ion gels maintained their solid-like integrity, irrespective of temperature; this could be attributed to photodimerization-induced chemical crosslinking of the micelle cores. The thermoreversible nature of the ion gel and the photoresponsive conversion from a temperature-responsive physical ion gel to a temperature-independent chemical ion gel enable facile processing at temperatures above *T*_gel_ and ensure the post-processing mechanical stability of the ion gel.

## Conflicts of interest

There are no conflicts to declare.

## Supplementary Material

RA-008-C7RA13181J-s001
