# Supplementary material for: Photocurable ABA triblock copolymer-based ion gels utilizing photodimerization of coumarin
Source: RSC Adv. 2018 Jan 17;8(7):3418–22. doi: 10.1039/c7ra13181j (PMC9077674; doi:10.1039/c7ra13181j)
Supplement: RA-008-C7RA13181J-s001 [file RA-008-C7RA13181J-s001.pdf]

Electronic Supplementary Material (ESI) for RSC Advances.
This journal is © The Royal Society of Chemistry 2018

Electronic Supplementary Information (ESI)

Photocurable ABA triblock copolymer-based ion gels utilizing photodimerization of coumarin

Ryota Tamate[1], Takeshi Ueki[2], Aya Mizutani Akimoto[3], Ryo Yoshida[3], Toshiyuki Oyama[1], Hisashi Kokubo[1] and Masayoshi Watanabe[1*]

[1]Department of Chemistry and Biotechnology, Yokohama National University, 79-5 Tokiwadai, Hodogaya-ku, Yokohama 240-8501, Japan.

[2]National Institute for Materials Science (NIMS), 1-1 Namiki, Tsukuba, Ibaraki, 305-0044, Japan.

[3]Department of Materials Engineering School of Engineering, The University of Tokyo, 7-3-1 Hongo, Bunkyo-ku, Tokyo, 113-8656, Japan.

[*]To whom correspondence should be addressed. E-mail: mwatanab@ynu.ac.jp. Phone/Fax: +81-45-339-3955

Experimental section

Materials

N-Isopropylacrylamide (NIPAm) was generously provided by KJ Chemicals (Japan) and recrystallized from toluene/*n*-hexane. Poly(ethylene oxide) (PEO, M_n = 35 kDa, polydispersity index (PDI) = 1.08) was purchased from Sigma-Aldrich (MO, USA) and purified by reprecipitation from toluene (a good solvent) and *n*-hexane (a poor solvent). *S*-1-Dodecyl-*S'*-(α,α'-dimethyl-α''-acetic acid)-trithiocarbonate (DDMAT) was purchased from Trylead Chemical Technology (China). 7-(2-Acryloyloxyethoxy)-4-methylcoumarin (coumarin acrylate, CA) was synthesized according to a previous report.[S1] 1-Butyl-3-methylimidazolium hexafluorophosphate ([C_4mim]PF_6) was purchased from Kanto Chemical (Japan). 2,2-Azobisisobutyronitrile (AIBN) was purchased from Wako Pure Chemical Industries (Japan) and recrystallized from methanol. Other reagents were used as received unless stated otherwise.

Polymer synthesis and characterization

The ABA triblock copolymer, P(NIPAm-*r*-CA)-*b*-PEO-*b*-(NIPAm-*r*-CA), was synthesized according to a previous report, with a slight modification.[S1] The PEO macro chain transfer agent (PEO-CTA) was synthesized according to a previous report.[S2] PEO-

CTA (1.50 g, 0.0420 mmol), NIPAm (4.28 g, 37.8 mmol) and CA (0.545 g, 1.99 mmol) were dissolved in 1,4-dioxane (25 mL). The initiator, 2,2′-azobis(2,4-dimethylvaleronitrile) (2.09 mg, 8.40 μmol) in 100 μL of 1,4-dioxane, was added to the solution, followed by bubbling with Ar for 30 min at room temperature. The reversible addition-fragmentation chain transfer (RAFT) polymerization was allowed to proceed at 65 °C for 3 h. The solution was diluted with acetone and purified by precipitation in *n*-hexane twice. The precipitate was dissolved in ethanol (50 mL) and an excess amount of AIBN (0.827 g, 5.04 mmol) dissolved in 50 mL of ethanol was added. After Ar bubbling for 30 min at room temperature, the solution was heated to 75 °C overnight to ensure complete removal of the terminal trithiocarbonate group. The polymer solution was concentrated using an evaporator and precipitated into diethyl ether twice. After drying under vacuum, the ABA triblock copolymer, P(NIPAm-*r*-CA)-*b*-PEO-*b*-P(NIPAm-*r*-CA), was obtained (yield: 2.56 g).

The PDI of the polymers was characterized by gel permeation chromatography (GPC), calibrated with PEO standards using dimethylformamide containing 0.01 M LiBr as an eluent (Fig. S1). The narrow PDI of 1.36 is indicative of successful RAFT polymerization; however, the slight high molecular weight shoulder as well as the low molecular weight peak indicate bimolecular termination and the presence of initiator-

derived polymer chains. The polymerization degree and number-averaged molecular weight (M_n) were calculated from ^{1}H NMR data (ECX 400, JEOL, Japan) (Fig. S2).

Sample preparation

All polymer solutions dissolved in ILs were prepared by a cosolvent method. An appropriate amount of the ABA triblock copolymer and [C_4mim]PF_6 were weighed and completely dissolved in dichloromethane (CH_2Cl_2), with stirring for at least 1 h at 50 °C. Then, CH_2Cl_2 was completely evaporated by heating the polymer solution in air and then under reduced pressure.

UV light source

A UV-LED spot light source, LC-L1V3 (Hamamatsu, Japan), with a peak wavelength of 365 ± 5 nm, was utilized as the UV light source for the UV-vis spectroscopy, dynamic light scattering and rheological measurements. The intensity of the UV light was fixed at 20 mW/cm^2.

UV-vis spectroscopy

Variation in the UV-vis spectra for the 0.1 wt% or 5 wt% ABA triblock

copolymer in [C_4mim]PF_6 under UV light irradiation was observed using a UV-vis spectrophotometer, UV-2700 (Shimadzu, Japan), at room temperature. A quartz cell with an optical path length of 1 mm was used for the measurement of the 0.1 wt% diluted polymer solution, whereas that with an optical path length of 0.1 mm was used for the measurement of the 5wt% concentrated polymer solution.

Dynamic light scattering (DLS)

Prior to the DLS experiments, the sample solution was passed through a 0.2 μm filter to eliminate any dust particles. DLS measurements were conducted on an Otsuka Electronic DLS-6500 (Otsuka electronics, Japan), equipped with an ALV correlator (ALV, Germany). A He-Ne laser (λ_0 = 632.8 nm) was used as the light source. Before the measurement, sample solutions were equilibrated for at least 30 min at different temperatures (20−70 °C), within an accuracy of ±0.1 °C. The second-order autocorrelation function was recorded at a scattering angle of 90° for 30 s, and the hydrodynamic radius (R_h) was calculated by using a cumulant method.[S3] Measurements were conducted in triplicate at each temperature.

Rheology

Rheological measurements were performed on an Anton Paar Physica MCR 102 (Anton Paar, Austria), with a standard cell for UV irradiation (P-PTD200/GL). Parallel-plate geometry, with a diameter of 25 mm and a gap spacing of approximately 0.2 mm, was used for all measurements. Temperature sweep measurements were conducted at a frequency of 1 rad/s and a strain amplitude of 2 %, with a heating rate of 1 °C/min over the range of 20−70 °C. Frequency sweep measurements were performed over a frequency range of 0.01−100 rad/s, with a strain amplitude of 1 %. Strain sweep measurements were performed at a frequency of 1 rad/s over a strain range of 0.1−1000 %. Stress relaxation experiments were conducted at a strain of 10 %. Rheological changes under UV irradiation (λ = 365 nm, 20 mW/cm^2) were investigated under the following constant conditions: frequency of 1 rad/s, strain of 1 %, and a temperature of 30 °C.

Photopatterning of the ion gel

A solution consisting of 5 wt% ABA triblock copolymer in $[C_4mim]PF_6$ dissolved in CH_2Cl_2 was cast on a glass slide, followed by evaporation of CH_2Cl_2 to form a cast ion-gel film. UV irradiation of the cast ion gel was conducted with a UV exposure apparatus equipped with a 2 kW ultrahigh-pressure mercury lamp (JP-2000-EXC, ORC

manufacturing, Japan) through a photomask. The intensity of the exposure was measured in the range of 320−390 nm with an ORC UV-331AP2. UV irradiation was performed for 1 h with an intensity of ca. 12 mW/cm^2. After irradiation, the ion gel was rinsed with chloroform and the non-irradiated area was removed.

Ionic conductivity

Ionic conductivity of the ion gel before and after UV irradiation was determined from impedance measurements with a 4192A LF Impedance Analyzer (Hewlett-Packard, CA, USA) over a frequency range from 5 Hz to 13 MHz, with an AC amplitude of 10 mV. The sample was filled between two stainless steel disk electrodes using a Teflon ring spacer (thickness = 2.0 mm; inner diameter = 8.3 mm). The sample was equilibrated at each temperature for at least 1 h before the measurement was performed.

Scheme S1 Synthetic scheme for preparation of the ABA triblock copolymer.

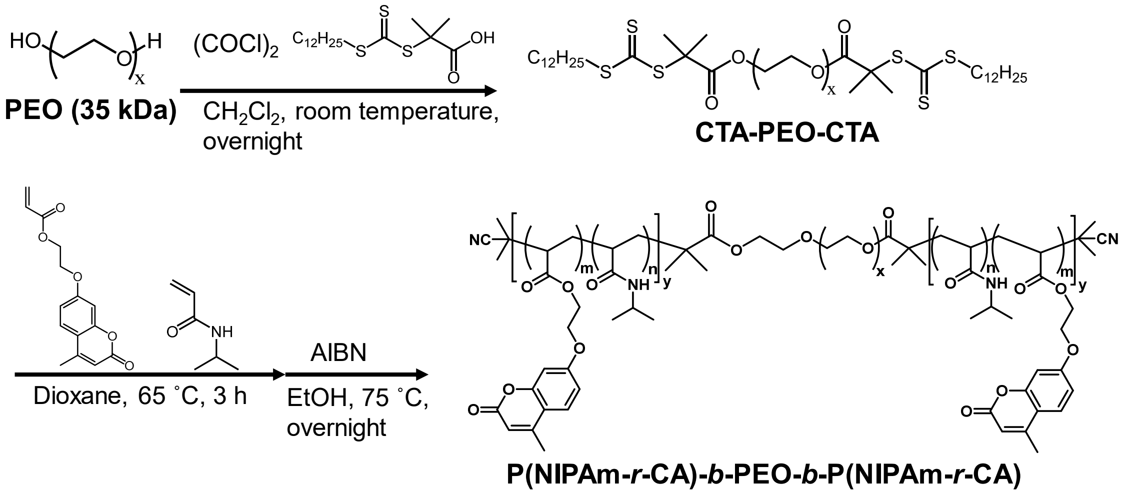

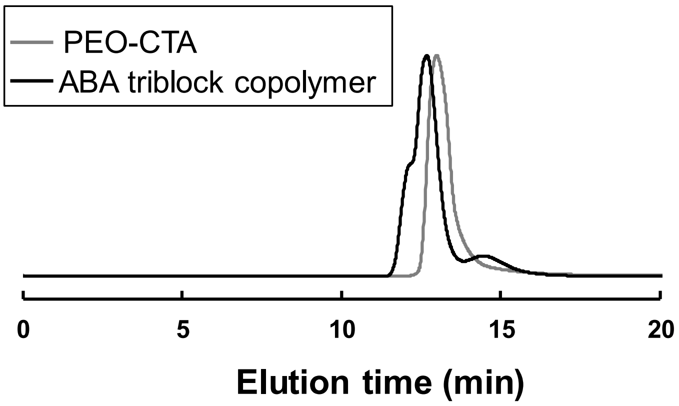

Fig. S1 GPC traces for PEO-CTA and the ABA triblock copolymer.

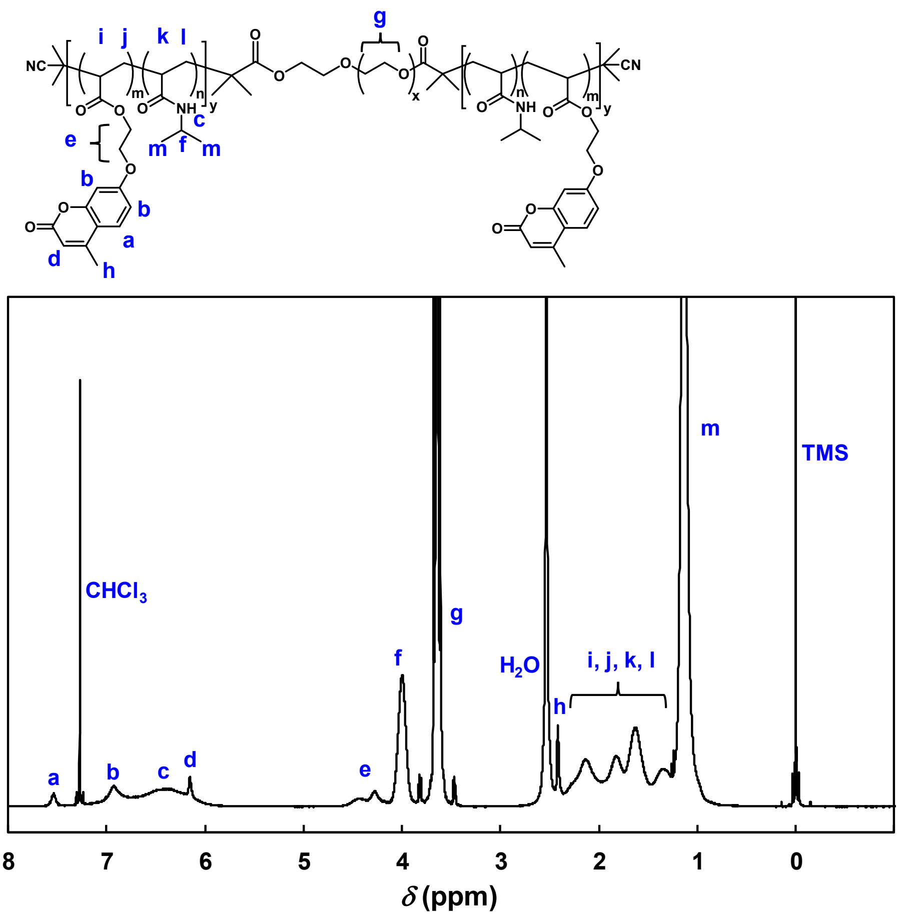

Fig. S2 ^{1}H NMR spectrum of the synthesized ABA triblock copolymer in $CDCl_3$.

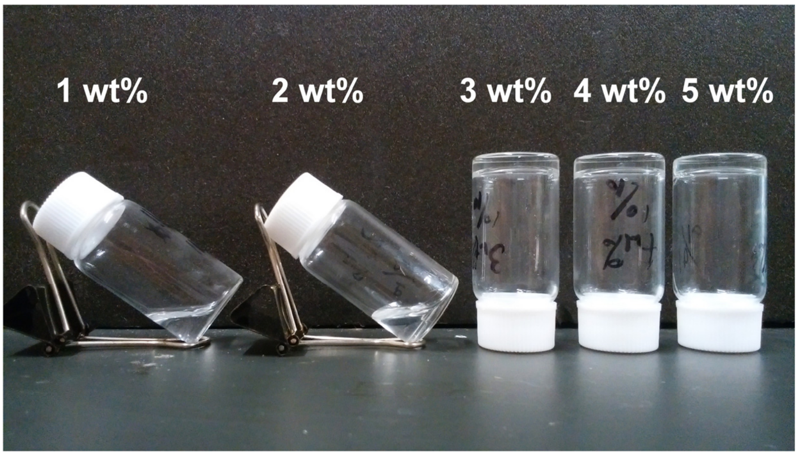

Fig. S3 Photograph of room-temperature qualitative inversion tests for the ABA triblock copolymer in $[C_4mim]PF_6$ with various polymer concentrations.

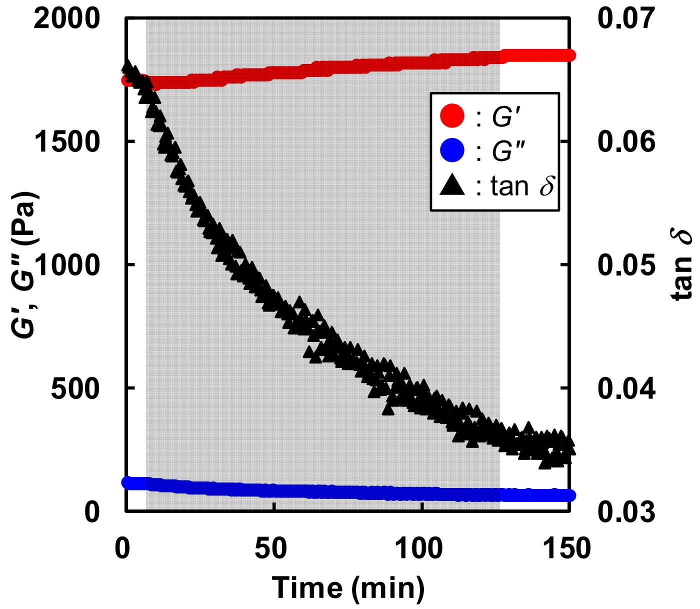

Fig. S4 Temporal variation in the storage (G') and loss (G'') moduli, as well as loss tangent (tan δ) for the 5 wt% ABA triblock copolymer in [C_4mim]PF_6 under UV irradiation, with a frequency of 1 rad/s and a strain of 1 %, at 30 °C. The time during which UV irradiation was applied is indicated by the gray area.

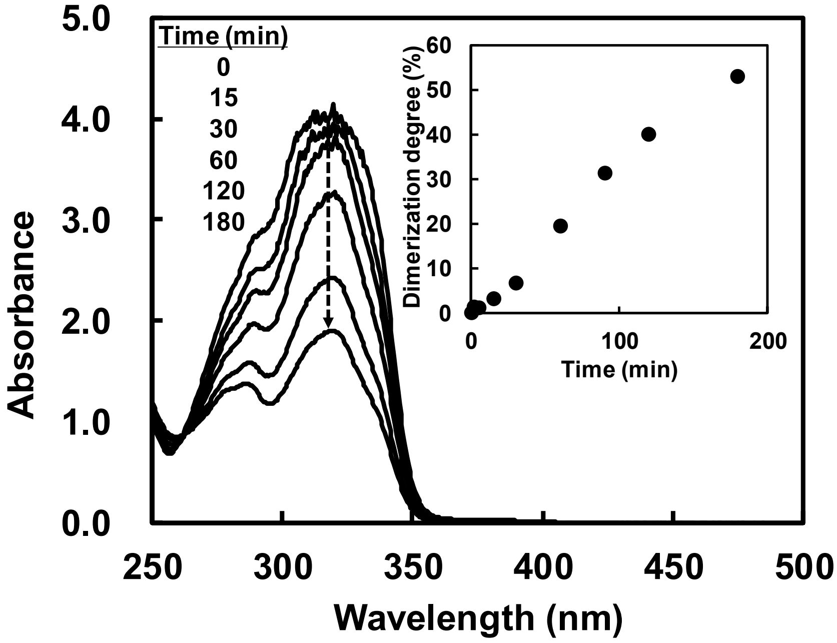

Fig. S5 Change in the UV-vis spectra of the 5 wt% ABA triblock copolymer ion gel under UV light at room temperature. (Inset) Time variation of the degree of dimerization.

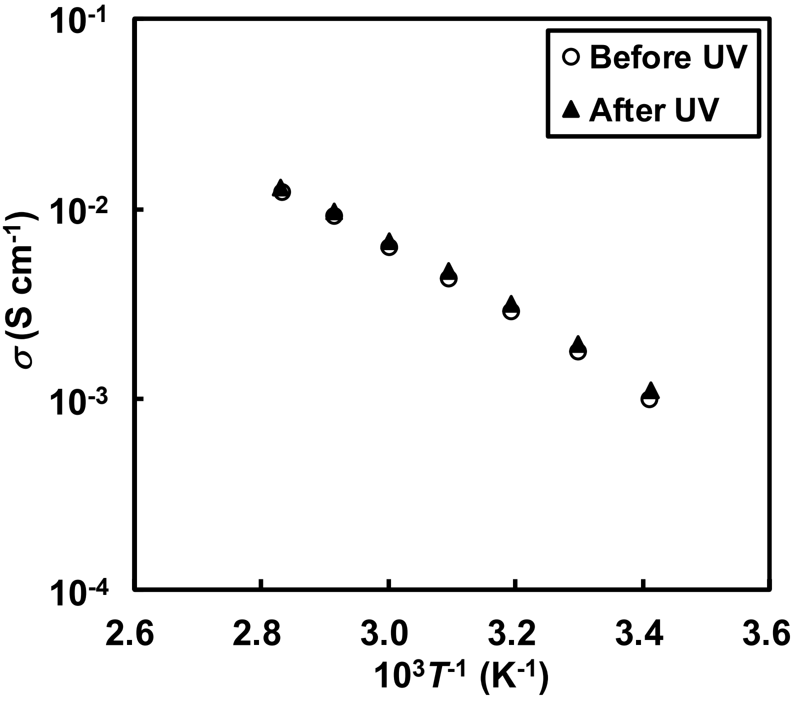

Fig. S6 Temperature dependence of the ionic conductivity before and after UV irradiation for the ABA triblock copolymer in [C_4mim]PF_6 (5 wt%).

Supporting References

S1 R. Tamate, T. Ueki, Y. Kitazawa, M. Kuzunuki, M. Watanabe, A. M. Akimoto and R. Yoshida, *Chem. Mater.*, 2016, **28,** 6401–6408.

S2 T. Ueki, Y. Nakamura, R. Usui, Y. Kitazawa, S. So, T. P. Lodge and M. Watanabe, *Angew. Chem. Int. Ed.*, 2015, **54,** 3018–3022.

S3 D. E. Koppel, *J. Chem. Phys.*, 1972, **57,** 4814–4820.
